# Sub-cellular *In-situ* Characterization of Ferritin(iron) in a Rodent Model of Spinal Cord Injury

**DOI:** 10.1038/s41598-018-21744-9

**Published:** 2018-02-23

**Authors:** A. R. Blissett, B. Deng, P. Wei, K. J. Walsh, B. Ollander, J. Sifford, A. D. Sauerbeck, D. W. McComb, D. M. McTigue, G. Agarwal

**Affiliations:** 1 Department of Biomedical Engineering, The Ohio State University, Columbus, OH 43210 USA; 2 Center for Electron Microscopy and Analysis, Department of Materials Science and Engineering, The Ohio State University, Columbus, OH 43210 USA; 3 The Center for Brain and Spinal Cord Repair and the Department of Neuroscience, The Ohio State University, Columbus, OH 43210 USA; 4 Biophysics Graduate Program, The Ohio State University, Columbus, OH 43210 USA

## Abstract

Iron (Fe) is an essential metal involved in a wide spectrum of physiological functions. Sub-cellular characterization of the size, composition, and distribution of ferritin(iron) can provide valuable information on iron storage and transport in health and disease. In this study we employ magnetic force microscopy (MFM), transmission electron microscopy (TEM), and electron energy loss spectroscopy (EELS) to characterize differences in ferritin(iron) distribution and composition across injured and non-injured tissues by employing a rodent model of spinal cord injury (SCI). Our biophysical and ultrastructural analyses provide novel insights into iron distribution which are not obtained by routine biochemical stains. In particular, ferritin(iron) rich lysosomes revealed increased heterogeneity in MFM signal from tissues of SCI animals. Ultrastructural analysis using TEM elucidated that both cytosolic and lysosomal ferritin(iron) density was increased in the injured (spinal cord) and non-injured (spleen) tissues of SCI as compared to naïve animals. *In-situ* EELs analysis revealed that ferritin(iron) was primarily in Fe^3+^ oxidation state in both naïve and SCI animal tissues. The insights provided by this study and the approaches utilized here can be applied broadly to other systemic problems involving iron regulation or to understand the fate of exogenously delivered iron-oxide nanoparticles.

## Introduction

Iron (Fe) is an essential metal involved in a wide spectrum of physiological functions, including oxygen transport, enzymatic reactions, energy production, protein synthesis and DNA repair^1–3^. Iron homeostasis is regulated by several factors including, but not limited to, iron confinement, its mineral size and composition, and its oxidation state. Ferritin is the major iron storage protein found in mammalian tissues comprising of a ~6–8 nm iron core with up to 4500 iron atoms typically in the form of ferrihydrite. The size, composition and distribution of the ferritin(iron) at the subcellular level is crucial to understand iron turnover in health and disease.

Iron assessment in pathology typically employs biochemical methods e.g. serum-ferritin levels, histochemical stains and/or immuno-labeling of tissue sections^4^. Oxidation state of iron in tissue sections can also be identified via Perls or Turnbull’s stains or by utilizing micro-focused X-ray beams^5,6^. Although these basic histology approaches are useful in rapidly evaluating iron content *in situ*, their limited spatial resolution cannot resolve subcellular iron distribution, which can lead to misleading results such as confounding iron-rich macrophages with biogenic magnetite^7,8^. In addition interpretation of *in-vivo* iron-assessment via approaches such as MRI^1,9^ would benefit by a comprehensive understanding of subcellular iron distribution.

High-resolution techniques such as transmission electron microscopy (TEM) have been useful to localize ferritin(iron) at the subcellular level in neurodegenerative^10,11^ and other pathologies^12,13^, in both rodent models^12^, and in human tissues^10,11,13^. Analytical TEM approaches^10,14–16^ have elucidated how the oxidation state of iron can differ in pathological vs. physiological tissues by analyzing the iron-rich particles isolated from various pathologies^17,18^. In these studies, it has been recognized that tissue digestion and extraction procedure not only destroys the cellular organization of the particles but can also influence the oxidation state of iron^14^. However, limited studies exist on subcellular mapping of the oxidation state of iron *in situ* in mammalian tissues. These include the perfusion-Perls and Turnbull’s method coupled with TEM imaging, which is primarily applicable to animal models^19^. Analytical TEM, while more versatile, has thus far been limited to neurodegenerative diseases^20^. Finally, although iron has been mapped *in situ* using magnetism-based microscopy^21,22^, the effects of particle size, crystal structure, density and oxidation state^23^ have not been adequately evaluated in these studies.

In this study, we examined ferritin(iron) in a well-characterized rodent model of central nervous system (CNS) injury, i.e., traumatic spinal cord injury (SCI)^24^, and how it differs from physiological ferritin *in situ* at the sub-cellular level. Biochemical histological stains were utilized to verify the presence of ferritin(iron) at the injury site (spinal cord) and in the spleen (an organ well-established for its role in iron metabolism^25^) from naive vs. SCI animals. A magnetism based approach, namely magnetic force microscopy (MFM), was utilized to analyze magnetic signal emanating from iron-rich lysosomes present in tissue sections *in-situ*. TEM was utilized to characterize the cytosolic and lysosomal ferritin(iron) density as well as lysosomal size. Electron energy loss spectroscopy (EELS), an analytical TEM technique, was used to evaluate Fe^3+^ percent in individual ferritin cores. Our results elucidate how ferritin(iron) distribution differs across injured and non-injured tissues at the sub-cellular level, which is not evident by routine biochemical staining. In addition, variations in the ferritin(iron) density in lysosomes could impact the magnetic signal which could be utilized in interpreting iron-status by MRI in pathological vs. physiological tissues.

## Results

### Biochemical Evaluation of Ferritin(Iron) in Injured and Non-Injured Tissues

**Figure 1:**
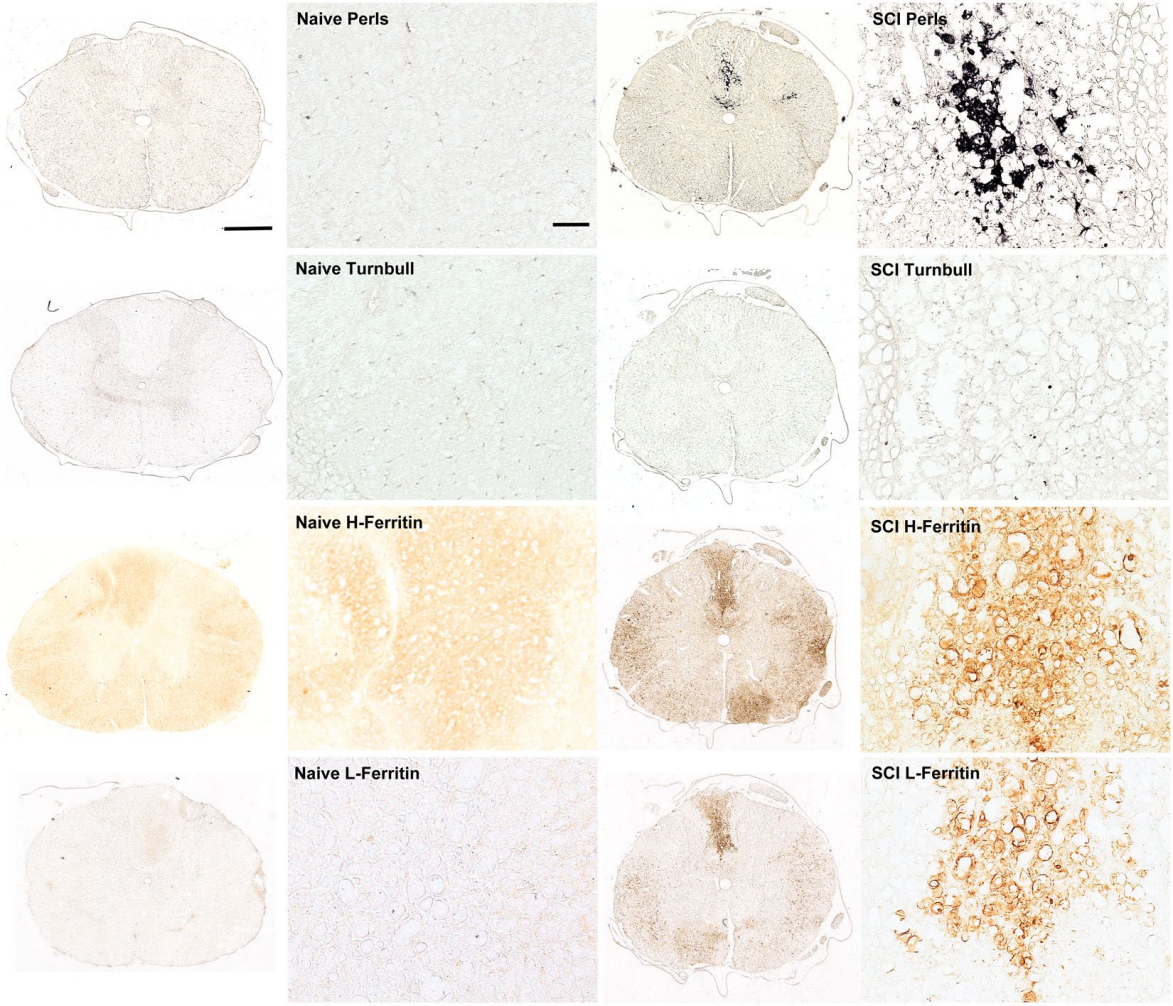
Biochemical analysis of spinal cord tissue shows Fe^3+^ (Perls) staining in injured (SCI) rats. No significant staining was observed for Fe^2+^ (Turnbull) in either naïve or SCI spinal cords. Immuno-histochemical (IHC) staining shows a basal expression of H-ferritin in naïve spinal cord but an increased expression of H and L-ferritin in SCI spinal cord in localized regions. Scale bars: 500 μm and 100 μm respectively.

Injured (SCI) and naive spinal cords were evaluated for the presence of iron using the Perls and Turnbull’s histochemical stains which indicate the presence of ferric (Fe^3+^) and ferrous (Fe^2+^) iron, respectively. We observed an increased Fe^3+^ iron content in injured spinal cords compared to naïve spinal cords (Fig. 1). Staining for Fe^2+^ in injured as well as naïve spinal cord tissues revealed no signal. Immunohistochemistry (IHC) of adjacent sections revealed the presence of both H- and L-ferritin in regions positive for Perls stain in injured spinal cord. As compared to L-ferritin, the expression of H-ferritin was more diffuse in the injured spinal cord and was also present in regions with little or no visible Fe^3+^ iron. Very little basal ferritin expression was detectable in spinal cord from naïve rats^26^.

**Figure 2:**
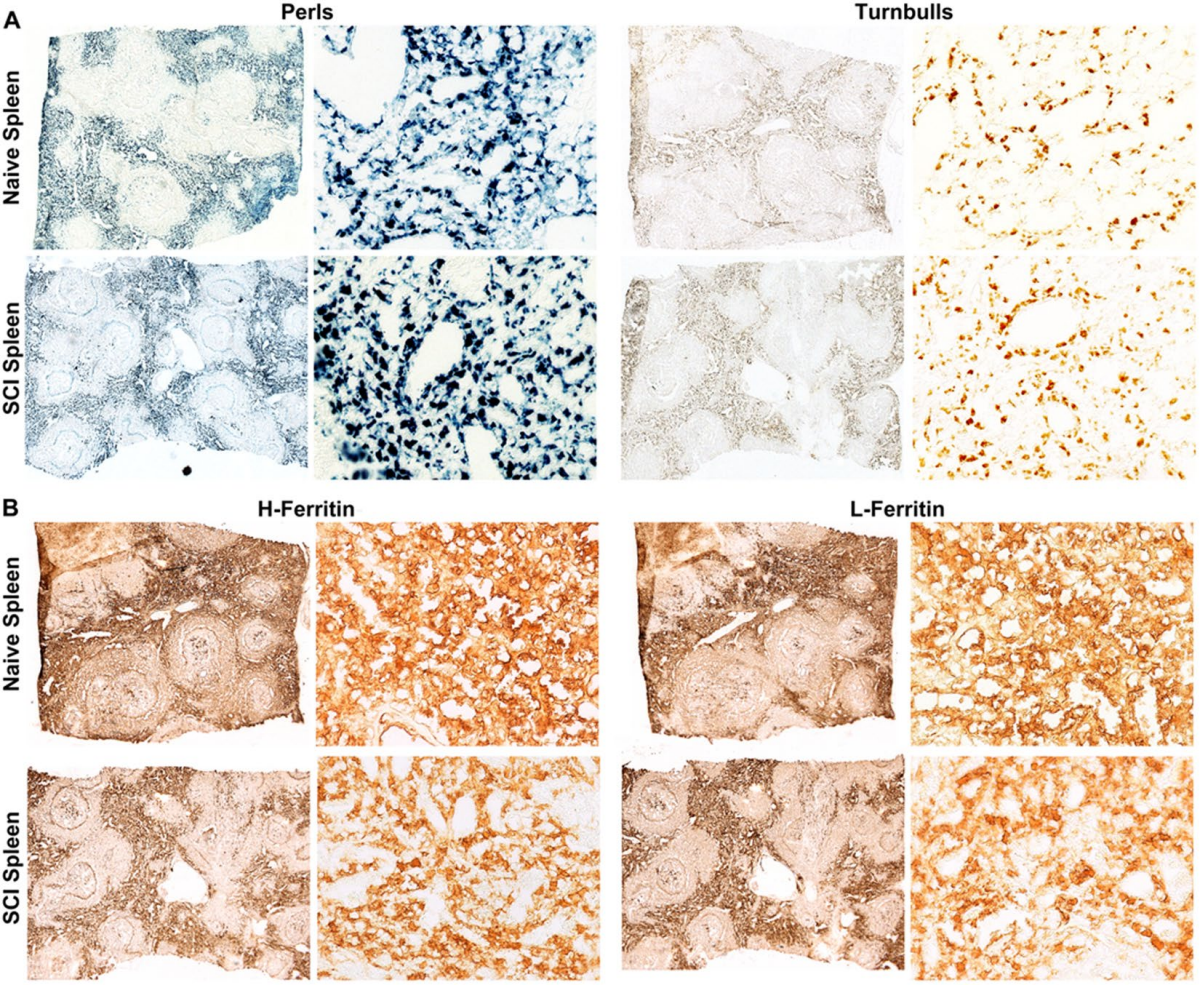
(**A**) Biochemical analysis of non-injured tissue (spleen) shows abundance of Fe^3+^ (Perls) as compared to Fe^2+^ (Turnbull’s) stain in both naïve and SCI rats. (**B**) IHC for H and L ferritin shows no difference in ferritin expression between spleens from naïve vs. SCI rats.

Spleen(s) harvested from SCI and naïve rats were also evaluated for the presence of ferritin(iron) using the biochemical stains described above. Spleen tissue from both naïve and SCI rats had abundant Fe^3+^ iron with no obvious differences in the level of Perls stain between the two groups (Fig. 2A). Turnbull’s staining showed a lower level of Fe^2+^ iron as compared to Fe^3+^ in the spleen sections with no significant difference between SCI and naïve animals. IHC of spleen tissue showed expression of L- and H-ferritin in regions that co-localized with the Perls stain (Fig. 2B). In contrast to injured spinal cord, the spatial expression of L- and H-ferritin was similar in spleens from injured and naïve rats. Thus our biochemical analyses revealed upregulation of Fe^3+^ iron primarily in the affected tissue (spinal cord) at sites of injury with no detectable changes in splenic iron storage in SCI animals compared to naïve.

### Magnetism Based Evaluation of Ferritin(Iron) in Injured and Non-Injured Tissues

**Figure 3:**
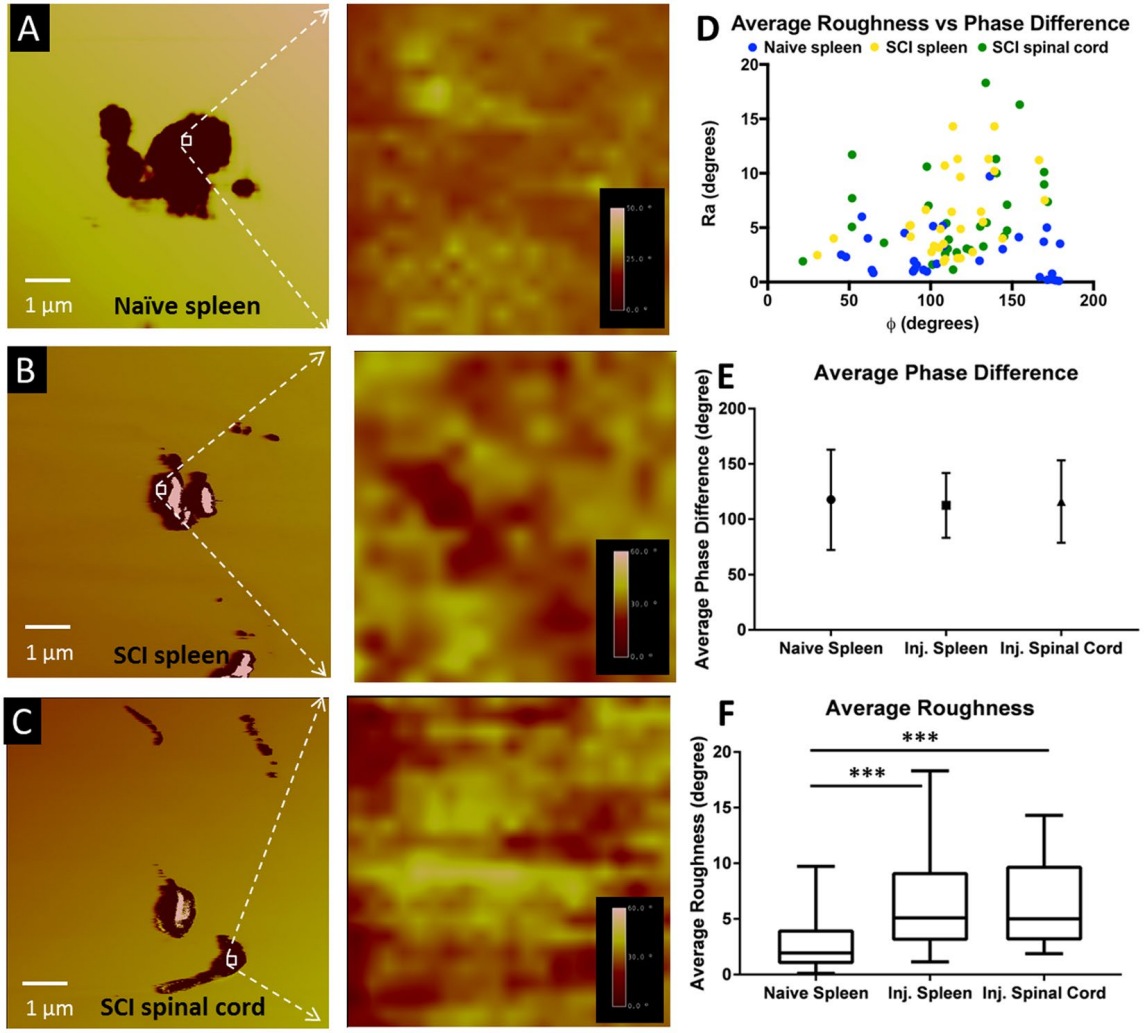
MFM phase images at a lift height, z = 30 nm for (**A**) naïve spleen, (**B**) spleen from SCI and (**C**) spinal cord from SCI rats. White square denotes the approximate location of a 0.04 μm^2^ ROI which was selected for roughness, R_a,_ analysis, as shown alongside each phase image. (D-F) Quantitative analysis of MFM phase ϕ and roughness R_a_ obtained from various regions in each sample type. ‘***’ Indicates p-value < 0.001.

As another measure of ferritin(iron) content, we evaluated the magnetic signal emanating from regions corresponding to Perls stained adjacent sections using magnetic force microscopy (MFM). As shown in Fig. 3A–C, long-range (z ≥ 30 nm) MFM signal (negative phase shift)^27^ could be detected in regions corresponding to Perls stain. The sizes of these regions exhibiting MFM signal corresponded to iron-rich lysosomes as demonstrated in our earlier study^21^. No MFM signal could be detected from monodisperse ferritin(iron) present in the cytoplasmic regions, consistent with our earlier study^21^. To evaluate if the MFM signal differed across tissues from naïve vs SCI animals, we analyzed the magnitude (ϕ) and average roughness (R_a_) of the regions exhibiting negative phase shift (Fig. 3D). Both ϕ and R_a_ showed regional variations in each sample, indicating heterogeneity in lysosomal composition. Interestingly while ϕ did not differ significantly across samples (Fig. 3E) (for z = 30) or for z = 40 or 50 nm (data not shown), R_a_ values were significantly higher for tissues from injured animals compared to the naïve spleen (Fig. 3D). An increased R_a_, could arise from several factors such as variations in the size, density, inter-particle interactions, oxidation state or crystal structure of the underlying magnetic nanoparticles^28^. We therefore performed ultrastructural TEM analysis on a subset of animals to characterize these features of ferritin(iron).

### Ultrastructural Evaluation of Ferritin(iron) Distribution in Injured and Non-Injured Tissues

**Figure 4:**
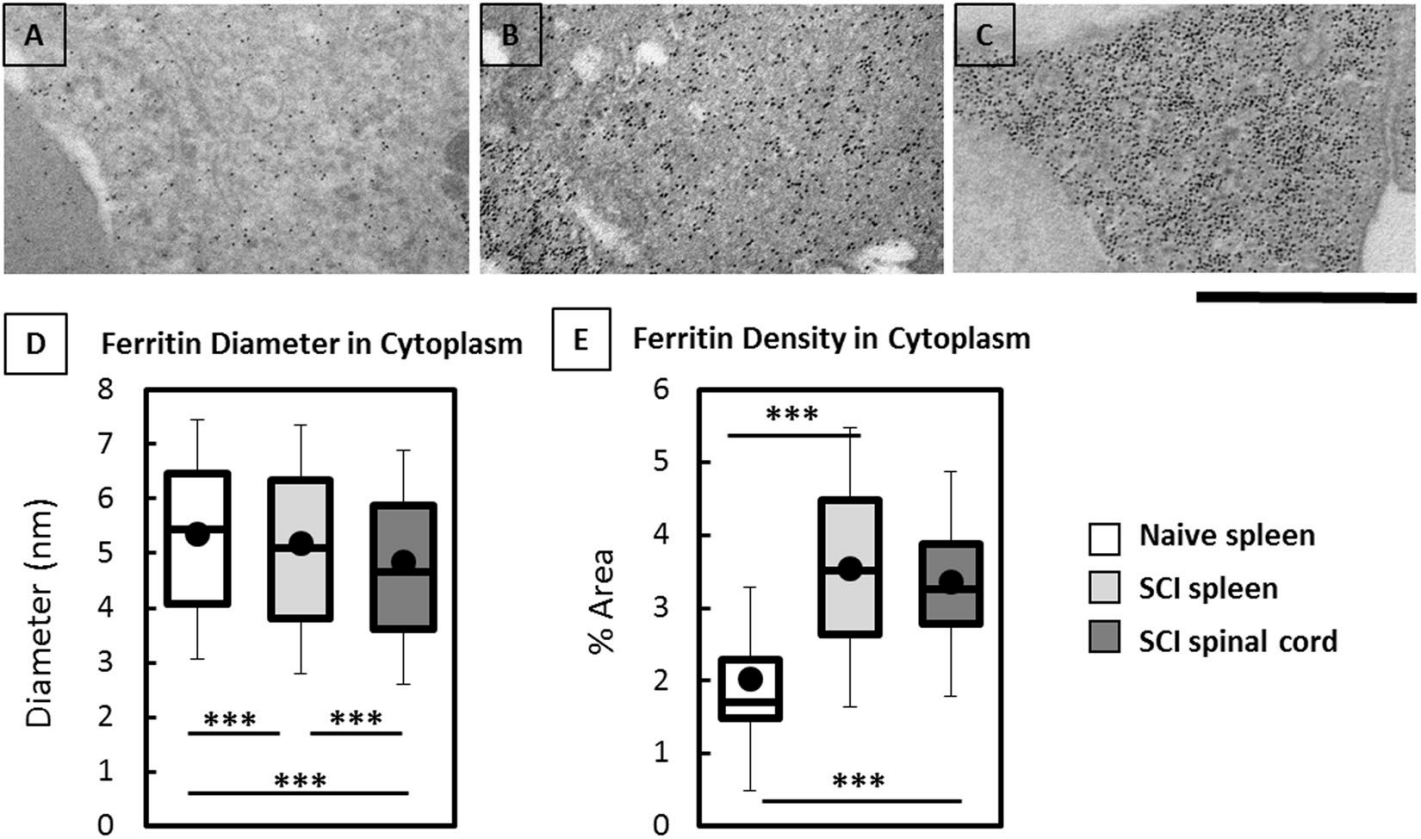
Ultrastructural characterization of the size and density of cytoplasmic ferritin(iron) (black dots) in macrophages using TEM imaging of (**A**) naïve spleen, (**B**) SCI spleen, and (**C**) SCI spinal cord. Scale bar is 500 nm. Box-plots display measured cytoplasmic ferritin diameter (**D**) and cytoplasmic ferritin density (**E**). ‘***’ Indicates p-value < 0.001.

Iron-loaded macrophages are prevalent in the acute spinal cord injury site and are maintained chronically as determined by Perls stain and basic IHC^27^. Thus, ferritin(iron) in spinal cord and splenic macrophages was analyzed using TEM to determine the morphology and distribution of cytoplasmic and lysosomal ferritin(iron). Uninjured spinal cords (from naïve animals) did not contain macrophages and thus were not included in these analyses. As shown in Fig. 4A–C, ferritin(iron) cores in the macrophage cytoplasm were monodispersed with an average diameter (~5 nm) comparable across all groups (Fig. 4D). Interestingly, the density of cytoplasmic ferritin(iron) significantly increased in the macrophages from both spinal cords and spleens of SCI rats compared to splenic macrophages in naïve rats (Fig. 4E). Our observations thus reveal an increase in cytoplasmic iron storage in systemic and CNS macrophage populations after SCI.

**Figure 5:**
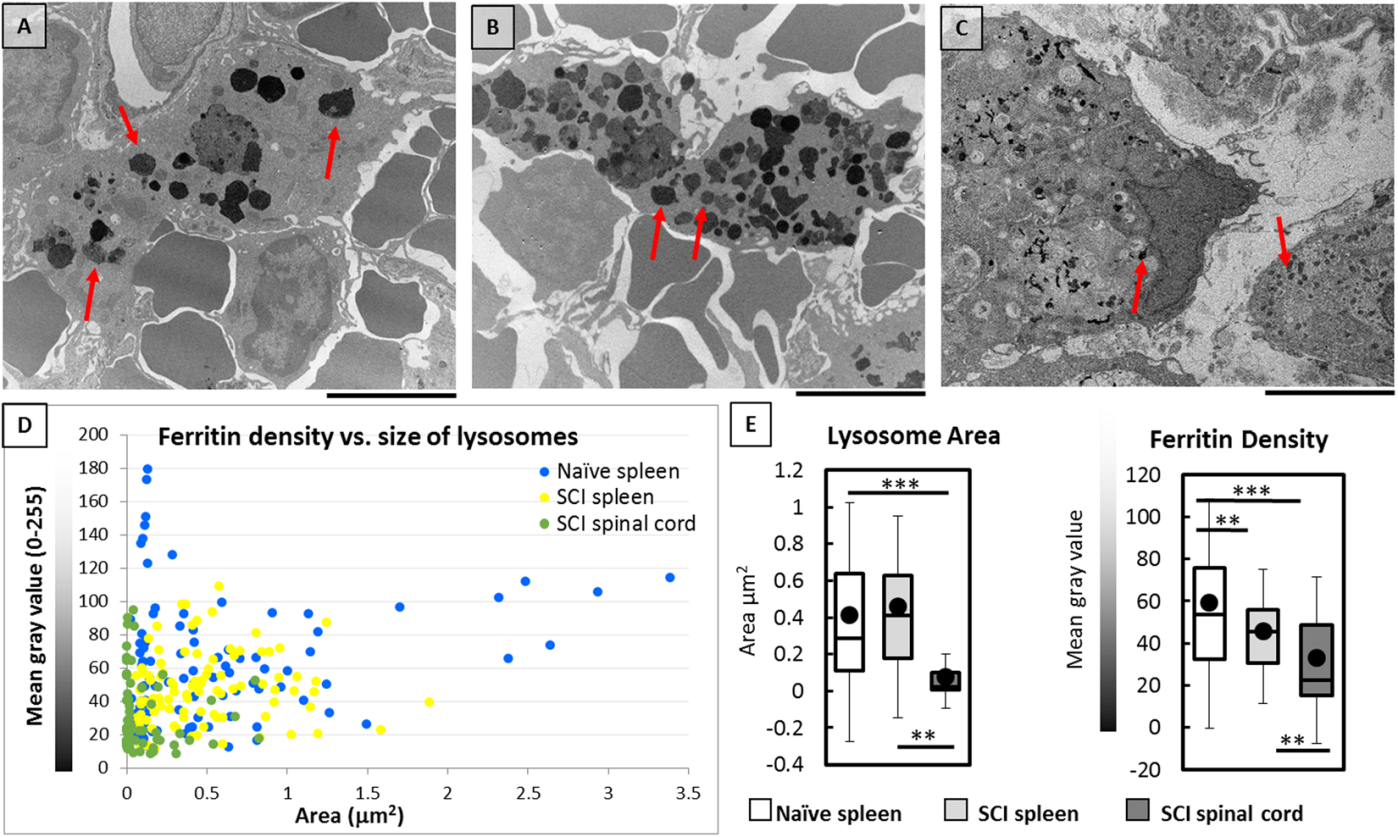
Ultrastructural characterization of the size and density of lysosomes containing ferritin(iron) (denoted by red arrows) in macrophages using TEM imaging of (**A**) naïve spleen, (B) SCI spleen, and (**C**) SCI spinal cord. Scale bar is 5 µm. (**D**) Scatter plot of ferritin(iron) density vs. lysosomal area, (**E**) box plots indicating lysosome area and ferritin(iron) density for each sample type. Low values of pixel gray values correspond to high ferritin(iron) density. ‘**’ And ‘***’ Indicate p-value < 0.01 and 0.001 respectively.

We next evaluated the lysosomal size and ferritin(iron) density in macrophage lysosomes for their potential correlation with the observed MFM signal. Lysosomes packed with ferritin(iron) could be identified as electron dense material in cells (Fig. 5A–C). Quantitative analysis of TEM images revealed that macrophages in naïve spleen had the broadest distribution of lysosomal size and density, compared to splenic and spinal macrophages from SCI animals (Fig. 5D). While there was no significant difference in average lysosome size in splenic macrophages from both animal groups, macrophages from the injured spinal cord site had significantly smaller lysosomes compared to splenic macrophages (Fig. 5E). Consistent with our observations regarding cytoplasmic ferritin(iron) density, the average density of lysosomal iron was significantly higher in the macrophages present in the spinal cord of SCI rats compared to those in the spleen of SCI or naïve animals (Fig. 5E). Splenic macrophages in SCI rats had a higher ferritin(iron) density compared to naïve rats. Thus SCI induced a shift in peripheral splenic macrophages to accumulate iron and ferritin, in a pattern similar to macrophages at the intraspinal injury site. Collectively these data extend our prior work^26,29^ by showing that the SCI site becomes populated with macrophages containing an abundance of cytoplasmic ferritin plus numerous small lysosomes with dense ferritin accumulation. In addition, our TEM analysis indicates that an increased R_a_ in the MFM data could arise due to the increased lysosomal ferritin(iron) density in tissues from SCI rats.

### Oxidation State of Ferritin (iron) in the Macrophage Cytoplasm and Lysosomes

**Figure 6:**
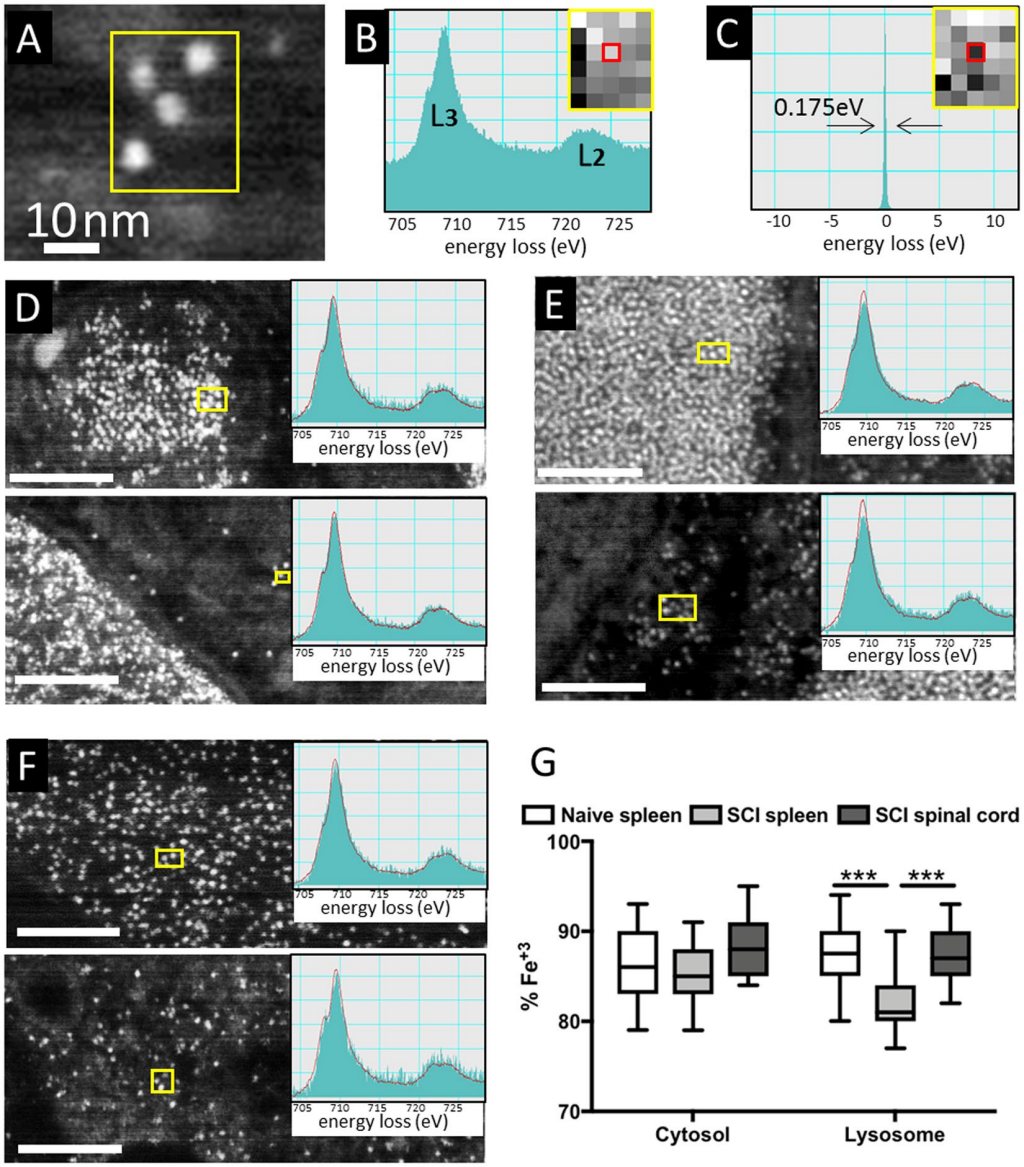
*In situ* EELS characterization of oxidation state of ferritin(iron) in the cytoplasm and lysosomes of macrophages. (**A**) STEM image of ferritin(iron) in naïve spleen. Corresponding high and low energy loss images of the same region are shown as insets in (**B**) and (**C**) respectively. (**B**) Spectrum showing Fe L_2,3_ edge from a selected pixel (red) in the high energy loss image. (**C**) Spectrum showing the zero-loss peak with energy resolution of 0.175 eV from a selected pixel (red) in the low energy loss image. (**D**–**F**) STEM image (inset: EELS spectra from a pixel in the selected region) from the lysosomes (top) and cytoplasm (bottom) of (**D**) naive spleen, (**E**) SCI spleen and (**F**) SCI spinal cord; scale bar: 100 nm. (**G**) Box plots showing percentage of Fe^3+^ in individual ferritin cores in the lysosome (**L**) and cytoplasm (**C**) of various samples as indicated. ‘***’ Indicates p-value < 0.001.

We examined the oxidation state of macrophage ferritin(iron) using EELS analysis on ultrathin spleen and spinal cord sections. EELS analysis was carried out with an energy resolution of 0.175 eV (Fig. 6A–C). Ferritin(iron) core could be identified as nanoscale bright dots in the lysosomes and cytoplasm in dark field STEM images (Fig. 6D–F**)**. EELS spectra was acquired and analyzed from individual ferritin(iron) cores. In concordance with the Perls and Turnbull’s data above, EELS showed that the major (>80%) iron content in ferritin particles of all samples was Fe^3+^ with a much smaller (<20%) percent of Fe^2+^. Within the cytoplasm, ferritin(iron) contained ~85% Fe^3+^ with no significant differences across samples. However, subtle differences in the percent of Fe^3+^ were detected in lysosomal ferritin (Fig. 6G). The oxidation state of iron in the lysosome of injured spinal cord closely resembled that of naïve spleen, while the percent of Fe^3+^ in the splenic lysosomes of injured animals showed a slight (~5%) decrease. Our results thus indicate that the oxidation state of ferritin(iron) was primarily Fe^3+^ in all tissues and may have little effect in modulating the R_a_ of the observed MFM signal.

## Discussion

We elucidate here how the ferritin(iron) content in a CNS injury model differs across tissues in injured and non-injured animals which is evident primarily at the ultrastructural level. As reported earlier^24^, routine histochemical staining confirmed an upregulation in Fe^3+^ (Perls iron stain) and L- and H-ferritin in the injured spinal cord at 21 days post-injury. No staining for Fe^2+^ (Turnbull’s iron stain) could be detected at this stage (except by the highly sensitive EELS technique). These observations are in agreement with a rodent model of kainite-induced brain injury where an upregulation of Fe^3+^ was detected as early as 1 week but Fe^2+^ positive cells could be detected only 2 months post-injury^30^. Further, no differences in the splenic iron or ferritin in the injured or naïve animal could be ascertained by basic histological stains.

Our *in situ* ultrastructural analysis revealed a significantly increased ferritin(iron) density in the cytoplasm and lysosomes of the macrophages present in the spleen and in the spinal cord of SCI rats as compared to naïve animals. This increase in macrophage ferritin(iron) content also correlated with an increase in serum ferritin for SCI rats as compared to naïve (data not shown), consistent with earlier reports^31^. Lysosome size and density is also understood to directly affect serum ferritin levels^31^ as well as cellular functions such as lysosome transport and inflammatory state of the cell^32,33^. It has been suggested that macrophages sequester excess iron into their cytosol which is then transported to lysosomes for degradation and recycling^34^. Thus SCI induced a shift in peripheral splenic macrophages to accumulate iron and ferritin, in a pattern similar to macrophages at the intraspinal injury site. A possible signal for post-SCI iron uptake by macrophages is SCI-induced TLR4 activation, which stimulates iron sequestration systemically in response to microbial invasion^35^. Although pathogens are absent from the spinal cord injury site, our prior work supports that post-SCI TLR4 activation promotes macrophage uptake of iron released by intraspinal hemorrhage and cell death^36^. The current work suggests that SCI also induces a systemic iron sequestration phenotype since splenic macrophages from SCI rats had a greater ferritin(iron) density compared to those from naïve animals. This may partially explain the post-SCI anemia noted in SCI individuals^37^, which is associated with SCI-related morbidities such as increased incidence and worse wound healing of pressure sores^38,39^.

The size of ferritin(iron) was ~ 5 nm in tissues from both naïve and injured animals. This measure is consistent with ferritin core sizes reported in various pathologies in earlier studies^10^. Larger (~10–200 nm) iron particles (biogenic magnetite), which have been identified in neurodegenerative brain tissue^40^, were not observed in our model of SCI. Further, our EELS analysis of the oxidation state of ferritin(iron) in our SCI model revealed that it is similar to physiological ferritin, consisting primarily of Fe^3+^ rich ferrihydrite. In contrast to our SCI model, pathological iron in prolonged brain injury^30^ and chronic neurodegenerative diseases^10,15^ has been reported to contain abundant Fe^2+^ rich magnetite. It has been postulated that overload and breakdown of the ferritin cage can serve as precursor for synthesis of biogenic magnetite^40^. In this regard, is interesting to note that the lysosomal ferritin(iron) density was highest in spinal macrophages of SCI rats, and the size(s) of these small ferritin(iron) dense lysosomes corresponds to the size of biogenic magnetite particles. It is thus plausible that formation of small, ferritin(iron)-dense lysosomes in infiltrating cells at sites of CNS injury could serve as precursor for formation of biogenic magnetite in chronic diseases. Taken together our results support the hypothesis that ferritin(iron) dense lysosomes in infiltrating cells consist initially of physiological ferritin which may undergo transformation to magnetite in chronic diseases.

We also characterized ferritin(iron) *in-situ* using MFM to evaluate the magnetic character of iron deposits. We could not detect MFM signal from monodisperse ferritin in the cytoplasm of all tissues examined, consistent with our earlier study^21^. Putative causes for this observation could be the low magnetic moment of monodisperse ferritins^41^ and the difference in the environment of cytoplasmic vs. lysosomal ferritin iron(core), e.g. presence of phosphates^42^, which may impact their magnetic properties^43,44^. MFM imaging could map ferritin(iron) dense regions of sizes corresponding to lysosomal areas in tissue sections, as demonstrated in our earlier study^21^. Long-range (z > 50 nm) MFM signal^27^ could be detected in regions corresponding to Perls stain with no significant difference in the magnitude of MFM signal across samples. However, the spleen and spinal cord from SCI rats were more heterogeneous in MFM signal than naïve spleen. Our TEM analysis supports that this magnetic heterogeneity is likely due to the increased lysosomal density and resulting inter-particle coupling^28^ of the ferritin(iron) core in tissues from SCI rats. Very few studies exist on magnetism based subcellular mapping of iron deposits in tissues. Our results demonstrate that ferritin(iron) dense lysosomes can also yield a magnetic response besides the biogenic magnetite particles reported in Alzheimer’s disease^16,22^.

We elucidate here how the ferritin(iron) content in a CNS injury model differs across tissues in injured and naive animals at the subcellular level even though no differences could be ascertained by basic histological stains in certain cases. For instance, no staining for Fe^2+^ (Turnbull’s iron stain) could be detected in the injured spinal cord at 21 days post-injury, despite positive identification by EELS revealing the high sensitivity of the EELS technique. Similarly, density of cytoplasmic and lysosomal ferritin(iron) as well as lysosomal size significantly varied across tissues. Very few studies exist on magnetism based subcellular mapping of iron deposits in tissues. These include biogenic magnetite particles reported in neurodegenerative diseases^42^ and other organisms^45^ or cells labeled with exogenously added magnetic nanoparticles^46^. Our results demonstrate that ferritin(iron) dense lysosomes naturally present in tissues also yield a MFM signal, which could be utilized for a more accurate assessment of iron status via approaches such as MRI^47^.

In summary, our results demonstrate the rich physiologically relevant data on iron status, obtainable with nanoscale methods that go beyond basic immunohistochemistry. These data provide greater insight into how iron is handled by different tissues following injury, which could in turn lead to better treatment strategies to combat systemic anemia or iron-induced cell death. The spinal contusion model used is clinically relevant as it mimics the most common injury suffered by humans. The approaches utilized here can be applied broadly to other systemic problems involving iron regulation or to understand the fate of exogenously delivered iron-oxide nanoparticles^48,49^ used in diagnostic or therapeutic applications.

## Materials and Methods

### Animal Procedure

All surgeries and postoperative care procedures were performed after approval by the Ohio State University Institutional Animal Care and Use Committee (IACUC). In addition, all methods were performed in accordance with the IACUC guidelines and regulations. Anesthetization of adult female Sprague Dawley rats was administrated via an 80 mg/kg ketamine and 10 mg/kg xylazine mixture. A laminectomy was performed at the thoracic T8 vertebral level to expose the underlying spinal cord, at which point a spinal contusion of moderate force (200 K dynes) was induced using the Infinite Horizons device (Precision Instruments). Following the injury, surrounding tissue was sutured and the skin closed with wound clips. Rats were administered 5 ml saline and placed in warm recovery cages. Antibiotics were administered for 5 days along with saline to support hydration. Bladder function was compromised; therefore, manual bladder expression was conducted twice each day until normal function returned. A group of rats not receiving SCI served as naïve controls. At 21 days post-injury, rats were deeply anesthetized (1.5× surgery dose) and then perfused transcardially with distilled water followed by fixation in 250 ml of 4% paraformaldehyde (PF) in PBS. For TEM studies the rats were perfused with a mixture of 4% PF and 2% glutaraldehyde in 0.1 M cacodylate buffer, pH 7.4. The entire spinal cord and spleen were extracted from these animals and segments of tissue from these organs were used for analysis as detailed in the following sections.

### Histochemical Iron Staining

Histochemical staining was performed tissues from n = 5 naïve or SCI animals. Segments (~2 mm in size) of the spinal cord (2 mm rostral to the epicenter of injury site) and spleen were embedded in Optimal Cutting Temperature (OCT) compound and snap frozen in liquid nitrogen. Blocks were transferred to −80 °C for subsequent freezing. Cross-sections were cut at 10 µm on a HM 505E (Microm) cryostat and mounted onto Superfrost Plus Microscope slides (Fisher Scientific). Sections were rinsed in distilled water 3 times to remove OCT compound, followed by several rinses in 0.1 M PBS and 15 minutes in 25% hydrogen peroxide in methanol. After several more rinses in PBS, tissue was permeabilized for 10 minutes in a 0.1% Triton x-100/PBS solution and the slides were again rinsed thereafter.

For staining of ferric iron (Perls Stain, Fe^3+^), a 2% potassium ferrocyanide solution in 2% hydrochloric acid (HCl) solution was incubated on tissue for 30 minutes. For staining of ferrous iron (Turnbull Stain, Fe^2+^), a 2% potassium ferricyanide in 2% HCl solution was incubated on tissue for 30 minutes. For all spinal cord sections, DAB enhancement (Vector Laboratories, SK-4100) was facilitated in order to visualize the stain. For DAB enhancement, the slides were rinsed in 0.1 M PBS 3 times for 5 minutes each, then incubated in 0.1% Triton x-100/PBS solution for 10 minutes followed by a quick rinse in 0.1 M in PBS. The slides were then incubated with DAB enhancement solution (4 ml water with 4 drops DAB, 2 drops buffer, 3 drops nickel, 3 drops H_2_O_2_) for 1 min and then rinsed with H_2_O, dehydrated in an ethanol series, cleared with xylene and coverslipped with Permaslip (Alban Scientific).

### IHC for ferritin

Immunohistochemical (IHC) labeling on adjacent tissue sections was performed to identify ferritin protein expression (rabbit anti-H-ferritin or rabbit anti-L-ferritin; Abcam). Briefly, sections were rinsed in 0.1 M PBS, blocked for nonspecific antigen binding using 4%BSA/0.3% Triton-100/PBS for 1 h and then incubated with primary antibodies overnight at 4 °C. The next day, sections were rinsed and incubated with secondary antibodies (goat anti-rabbit IgG; Vector) for 1 h. After rinsing, endogenous peroxidase activity was quenched using a 4:1 solution of methanol/30% hydrogen peroxide for 15 min in the dark. Sections were treated with Elite avidin– biotin enzyme complex (ABC; Vector Laboratories) for 1 h. Visualization of labeling was achieved using DAB substrates (Vector Laboratories). Sections were rinsed, dehydrated, and coverslipped with Permount (Thermo Fisher Scientific). All slides were imaged using a Zeiss Axioshop 2 Plus microscope equipped with a Sony 970 three-chip colored camera.

### Magnetic Force Microscopy

Adjacent sections as that used for histochemical staining and IHC were used for magnetic force microscopy (MFM) as described earlier^21^. MFM studies were performed on tissues from n = 5 animals per group. The OCT embedded tissues were cryo-sectioned into 5 μm thick sections on poly-lysine coated glass coverslips, rinsed three times with ultrapure water and allowed to dry overnight in ambient air^50^. The glass coverslips containing the tissue sections were adhered to a metallic stub and mounted onto the JV scanner of a Multimode AFM equipped with a Nanoscope IIIa Controller (Bruker) and a reflected light module mounted over the AFM head. MFM was performed using pre-magnetized high moment (HM) MFM probes (ASYMFMHM, Asylum Research) in the tapping mode. Topographic (height) images (in main mode) and phase images (in lift mode) were recorded using the interleave scan feature of the Nanoscope software. MFM scans at a lift height (z) of 30, 40 and 50 nm were obtained from several areas corresponding to iron-rich regions as identified from adjacent sections with Perls stain. MFM phase signal was measured from regions showing a negative phase shift, using the section analysis feature of the Nanoscope software Analysis 1.5. The average roughness, R_a_ (the variation in phase across a region), was determined by selecting ROIs of sizes 0.03 to 0.04 μm² within a region of negative phase shift for lift height of z = 30 nm using the Nanoscope software. R_a_ was ascertained for at least n = 6 different regions from each animal for each tissue type. Statistical analyses was performed on n = 30 R_a_ values obtained from naïve vs. SCI animal groups for each tissue type.

### Transmission Electron Microscopy

A subset of animals (n = 3) were utilized for TEM studies. Lesion epicenters from spinal cord injury sites (T8) were dissected into <1 mm segments, immersion-fixed overnight at 4 °C, and placed in 0.2 M cacodylate buffer for storage. Segments of the spleen tissue were processed similarly. Tissue was processed for TEM analysis, beginning with a 1-hour immersion in 1% osmium tetroxide. Samples underwent a graded ethanol dehydration series (30–100%) followed by an exchange into acetone transition solvent and subsequent infiltration series with an epoxy resin mixture (Spurr’s formulation). Following polymerization overnight in a 60 °C oven, resin blocks were semi-thin sectioned at 750 nm and stained with Methylene Blue-Azure II and Basic Fuchsin stain in order to identify regions of interest (ROI) within samples. Blocks were trimmed down to ROIs, and 40 nm thin sections were cut on a Leica Ultracut UCT ultramicrotome (Leica-Microsystems) using a Diatome Ultra knife and collected on 200 mesh copper grids. No staining after thin-sectioning was done. For conventional imaging, sections were examined on a JEOL JEM-1400 (JEOL, Peabody, MA) operating at 80 kV and digital micrographs were captured on an Olympus Veleta camera.

For analysis of ferritin particle diameter, TEM images were converted into an 8-bit format using the NIH Image J software. Regions of interest (ROIs) were selected within macrophages and processed through a bandpass filter where large structures were filtered down to 40 pixels and small structures up to 4 pixels. Ferritin iron could be easily distinguished as electron-dense particles, in filtered and thresholded ROIs. The area for each particle was assessed by using the “analyze particle” feature provided by Image J. Particle diameter was calculated by approximating the measured area to a circle. To quantify ferritin density within the macrophage cytosol, the same micrographs were analyzed using the “percent area” feature provided by Image J. Macrophage lysosomes were hand-traced and evaluated for area and pixel density as described earlier^21^.

### Electron Energy-Loss Spectroscopy (EELS)

STEM images and electron energy-loss spectroscopy (EELS) spectra were recorded on an image Corrected Titan3^TM^ G2 60–300 S/TEM equipped with Gatan imaging filter (GIF) system. EELS spectra were acquired with 0.025 eV/channel energy dispersion and an energy resolution of 0.175 eV. Energy resolution was measured from the full width at half maximum of the zero-loss peak (see Fig. 5A). High-loss and low-loss spectra were collected simultaneously from the same area of interest by a dual-EELS spectrometer. Spectra were energy calibrated. High-loss spectra were then background subtracted using a power-law background extrapolation. The multiple-inelastic scattering effects were removed using the Fourier-ratio technique. The percentage of Fe^3+^ iron present in each iron core was determined by fitting reference spectra to the Fe L-edge of the high-loss spectra. The four single valence reference mineral spectra used in the fitting procedures were: Haematite, Fe-Orthoclase, Hedenbergite and Hercynite^51^. Multiple Linear Least-Square (MLLS) fitting of reference spectra was applied to measure the content of Fe^3+^ and Fe^2+^ for each spectra^52^. At least n = 18 ferritin(iron) cores were analyzed in the lysosome and in the cytoplasm of macrophages in each sample type.

### Statistical analysis

Statistical analysis was conducted on data sets obtained by analyzing images acquired from the naïve vs. SCI animals. Data analysis was carried out using GraphPad Prism 5.0. Data were compared using one-way analysis of variance followed by Bonferroni’s Multiple Comparison test. Data were considered significant when p < 0.05. All data are represented as mean + /− SD.

### Data Availability

The datasets generated during and/or analysed during the current study are available from the corresponding author on reasonable request.
